# A Double Barrier Technique in Surgical Closure of Oroantral Communication

**DOI:** 10.7759/cureus.31671

**Published:** 2022-11-19

**Authors:** Supriyo Pal, Keerthana Rao, Nelson Sanjenbam, Nikesh Thounaojam, Rajkumari Geeta, Hiroj Bagde

**Affiliations:** 1 Department of Oral and Maxillofacial Surgery, Sri Siddhartha Academy of Higher Education, Tumkur, IND; 2 Department of Oral and Maxillofacial Surgery, Jawaharlal Nehru Institute of Medical Sciences, Imphal, IND; 3 Department of Periodontology, Sri Siddhartha Academy of Higher Education, Tumkur, IND; 4 Department of Dentistry, Manipur Health Service Manipur University, Imphal, IND; 5 Department of Periodontology, Rama Dental College and Research Centre, Kanpur, IND

**Keywords:** case report, maxillary sinus, guided tissue regeneration (gtr) membrane, platelet rich fibrin (prf), oro antral communication (oac)

## Abstract

Routine minor surgical procedures in the maxillary premolar or molar region often heal without any repercussions; however, some may culminate in an unintentional opening into the maxillary sinus, leading to the formation of oroantral communication. It is, therefore, imperative for a surgeon to recognize it and treat it sequentially to avoid long-term complications. This case report highlights a flapless double membrane closure of oroantral communication (OAC) with platelet-rich fibrin (PRF) and guided tissue regeneration (GTR) membranes and its edge over conventional methods.

## Introduction

Oroantral communication (OAC) is a pathologically blind tract lined with epithelial tissue that communicates the maxillary sinus with the oral cavity. Extraction of maxillary molars, implant procedures, cysts, tumors, and, in certain cases, osteomyelitis and trauma are the common etiologies of OAC [1].

The success rate for immediate repairs of the acute oroantral defect is consistently high and approaches 95%; however, it drops to 67% in situations of delayed closure. The existence of sinus disorders plays a crucial part in the healing process [2]. To avoid post-operative comorbidities such as sinusitis and oroantral fistula (OAF), the fistula must be excised and the flap advanced to completely close the OAC. Single, double, or triple-layer flaps with autogenous, allograft, and alloplastic materials and stabilizing the graft with sutures are some of the modalities used to close OAC [3].

In our case, we elected to do a flapless double membrane closure of OAC with platelet-rich fibrin (PRF) and guided tissue regeneration (GTR) membranes, as PRF is a dependable source for extracting growth factors from patients' blood without the use of additives [4]. PRF releases transforming growth factor β1, platelet-derived growth factor, vascular endothelial growth factor, and glycoproteins such as thrombospondin. They boost fibroblast proliferation and increase vascularization without causing an immune reaction or posing an infection risk, and guided tissue regeneration (GTR) membrane has high biocompatibility, contributes to bone regeneration, and acts as a favorable passive barrier [5].

## Case presentation

**Figure 1:**
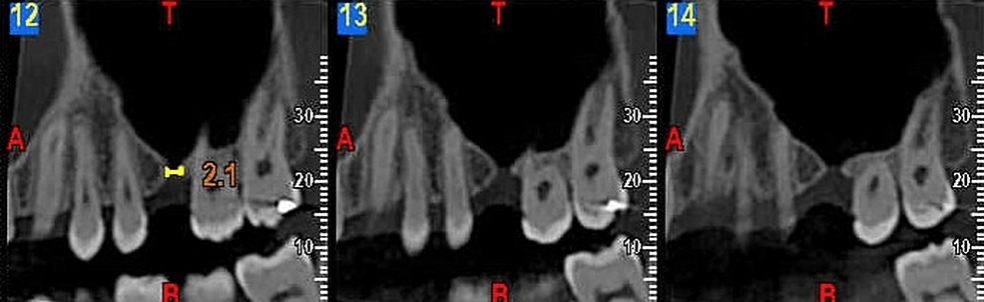
CBCT imaging of the lesion

A 22-year-old male patient reported a chief complaint of pain in the upper right back tooth region and water discharge and regurgitation of food from the nose while eating at Sri Siddhartha Academy of Higher Education. The informed consent was taken with an ethical clearance number of IEC 2022/1111. The patient underwent an extraction in the upper left back tooth region 4-5 years ago, following which he suffered from frequent colds and regurgitation of water from the nasal cavity. A comprehensive intraoral clinical examination was performed with tooth number 26. All of the inspection findings were validated by the Valsalva maneuver and mouth mirror fogging test. An intraoral periapical radiograph in relation to 27 was taken along with a cone beam computed tomography (CBCT) imaging (Figure 1), which showed a breach in the antral floor.

**Figure 2:**
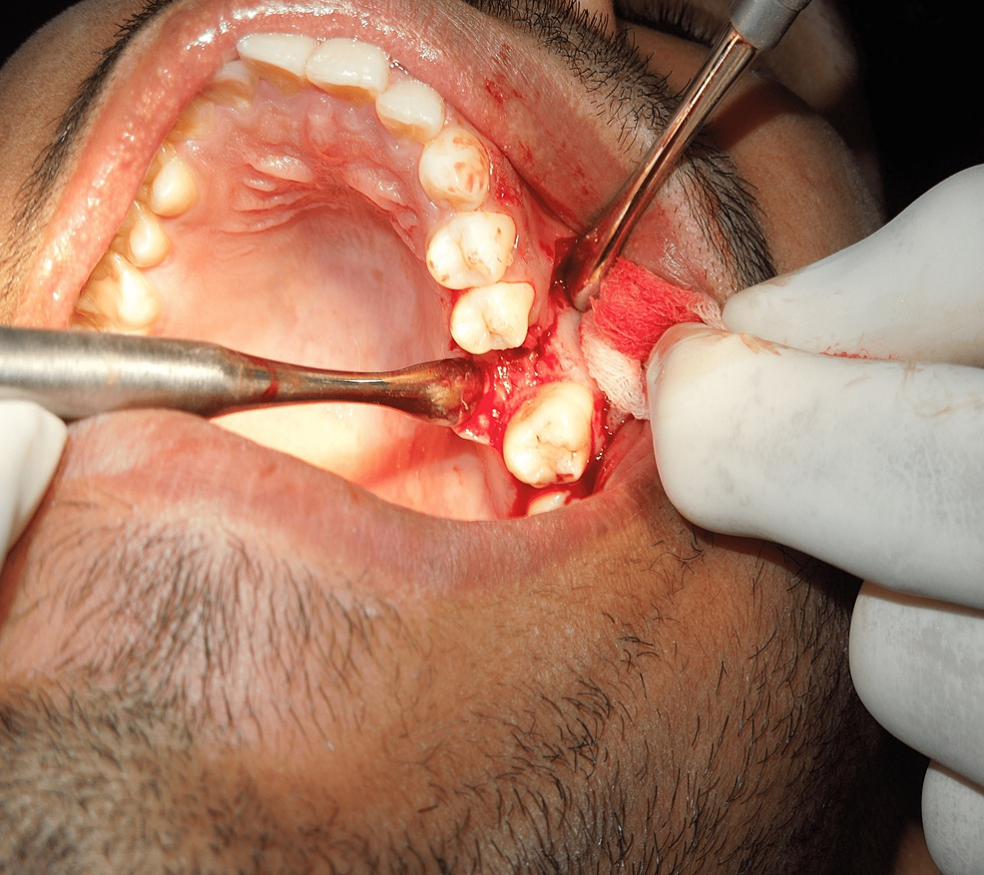
The fistula was identified and excised completely

After the confirmation of OAF, the following treatment plan was executed. The surgical site was cleaned with povidone-iodine and irrigated with saline. A posterior superior alveolar, middle superior alveolar, and greater palatine nerve block were given at the operative site with 2% lignocaine hydrochloride and 1:80,000 adrenaline. A cribriform incision was made extending from 25 to the distal end of 27, with an anterior releasing incision till the vestibular region with respect to the 25 regions. A triangular mucoperiosteal flap was elevated. The fistula was identified and completely excised with 15 C (Figure 2).

**Figure 3:**
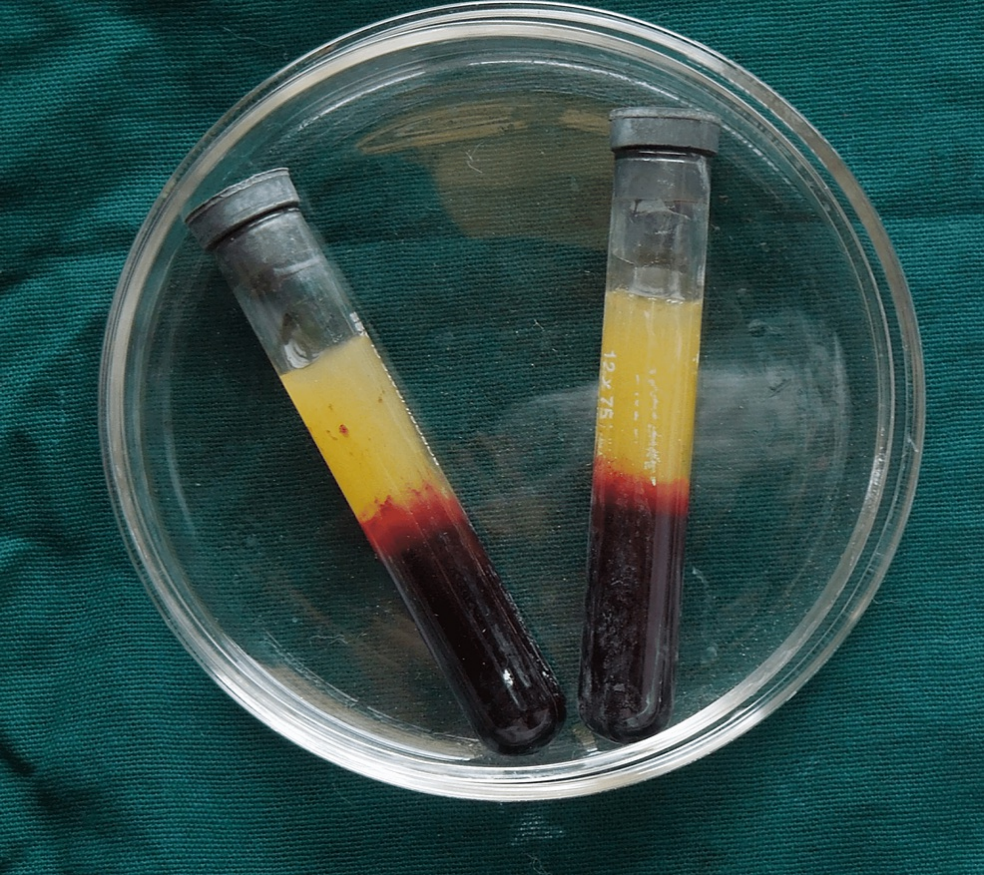
The PRF separated from the blood resides prepare PRF membrane

For tissue enhancement, autologous PRF and guided tissue regeneration (GTR) collagen membranes were used. Autogenous platelet-rich fibrin membrane was freshly prepared: 10 ml of blood was drawn from the patient and collected in glass-coated plastic tubes without anticoagulants, following which it was centrifuged at 3000 rpm for 12 minutes in a centrifuge machine. The PRF was then separated from the blood and transferred to a PRF box, and compressed with a slab to obtain a PRF membrane (Figure 3).

**Figure 4:**
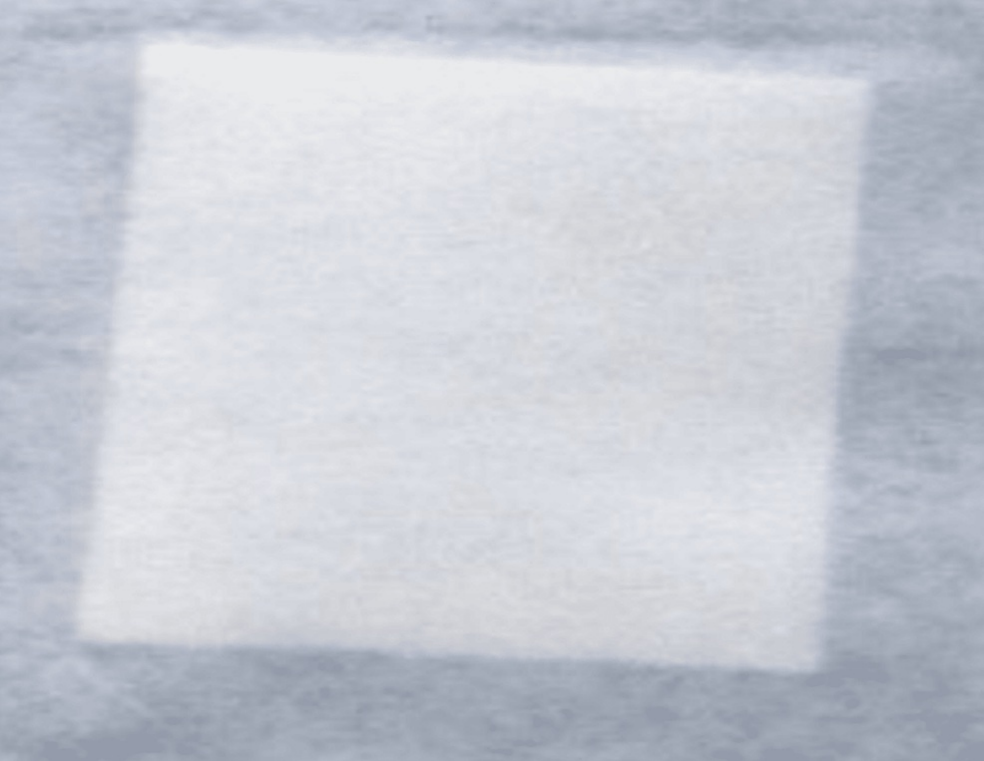
GTR collagen membrane

A commercially available periocolR was used as the GTR collagen membrane (Figure 4). 

**Figure 5:**
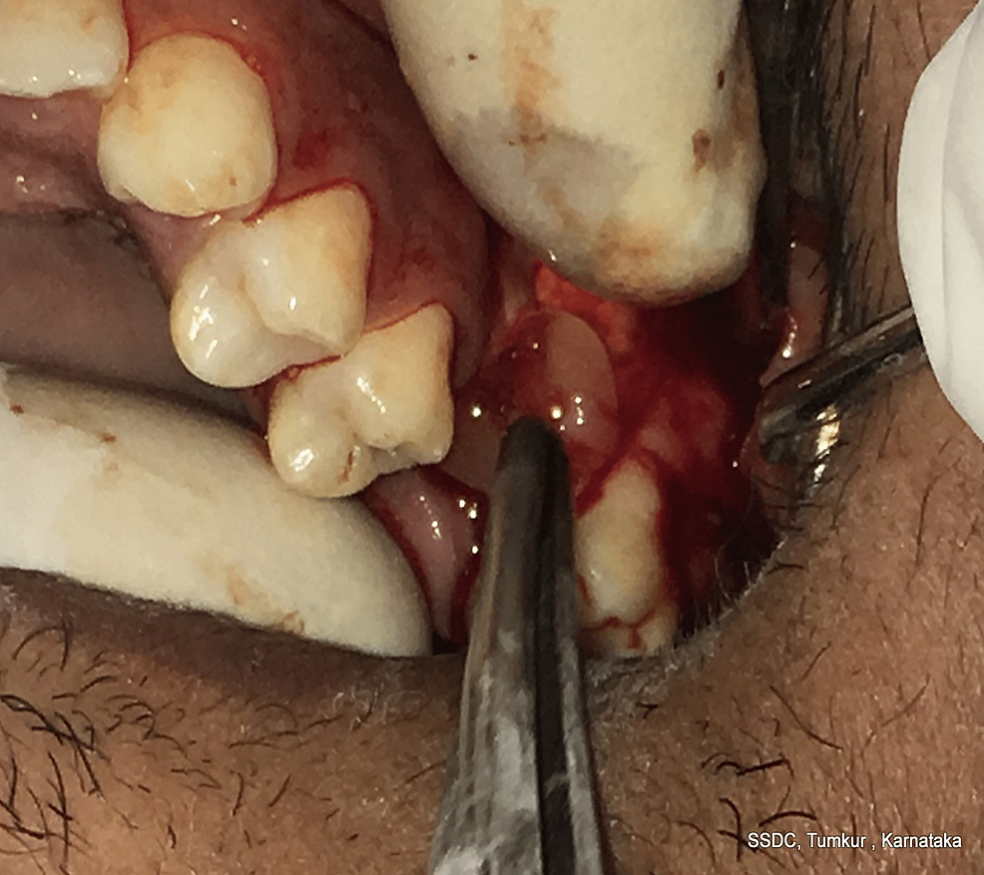
Placement of PRF membrane

After the membranes were obtained, the socket was irrigated with normal saline and dried. The PRF membrane was first placed over the socket, and the excess membrane was tugged into the socket between the Schneiderian membrane and the overlying bone (Figure 5).

**Figure 6:**
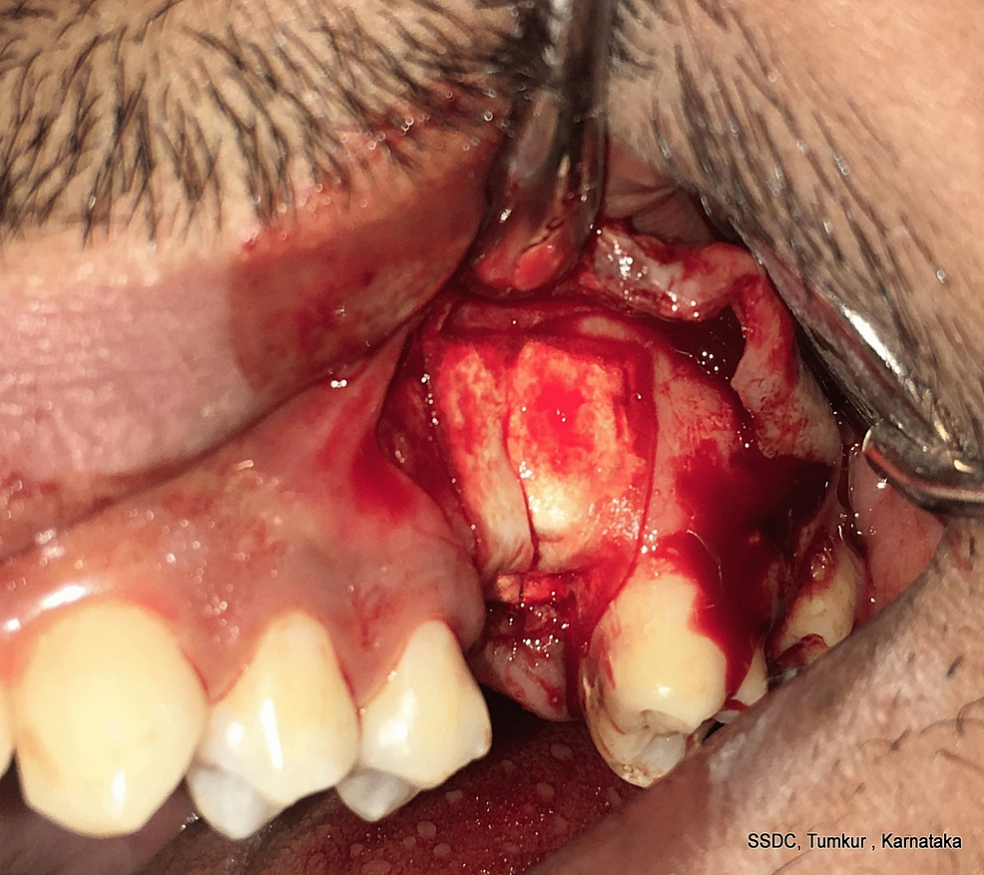
Placement of the GTR membrane over the PRF membrane

**Figure 7:**
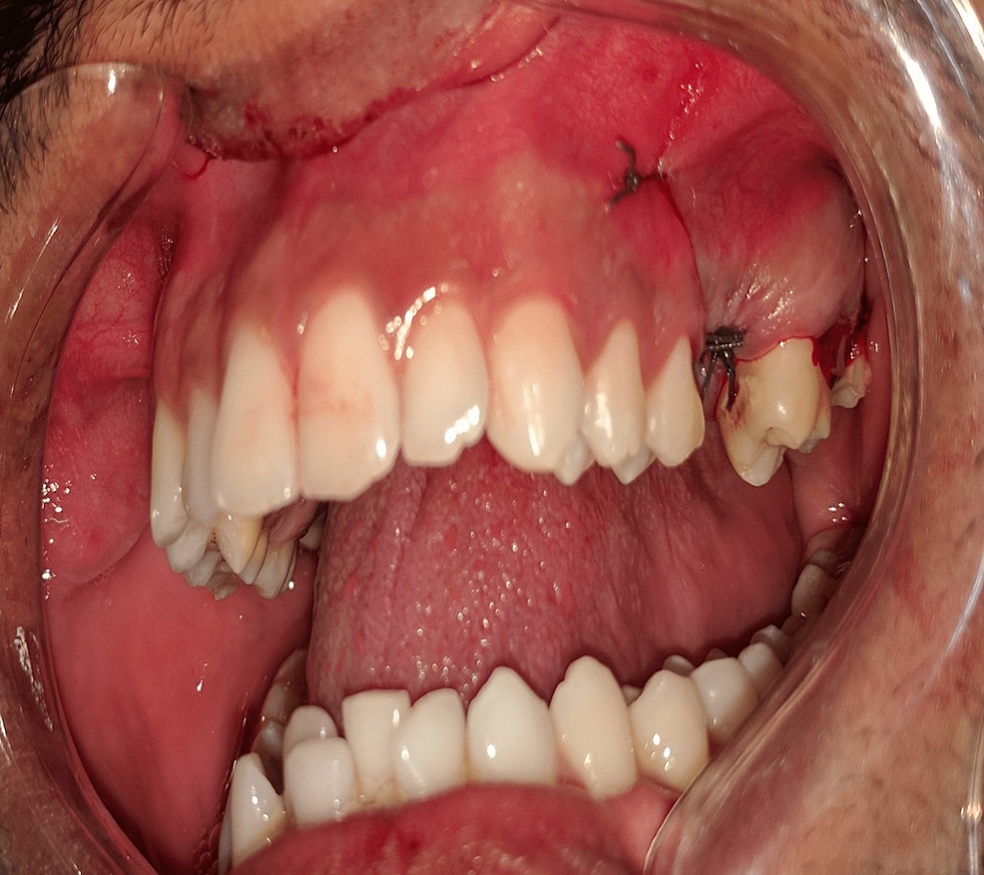
Suturing is done for double membranous sandwich closure of OAC

The GTR collagen membrane was then placed over this and sutured, making it a double membranous sandwich closure of the OAC (Figures 6, 7).

A CBCT was taken during the follow-up of three months, which revealed a thin cortical bone formation in the antral wall with respect to tooth numbers 26, 27, and 28. Following surgery, it's important to drink plenty of fluids, avoid eating anything crunchy, and stick to soft meals. To prevent injury or infection at the location of the operation, patients must consume meals and liquids on the opposite side. One should refrain from engaging in strenuous physical activity until the healing process is finished. Patients should wait at least seven days after surgery to contact the surgical site with their tongue. For at least two weeks, sneezing with the mouth closed should be avoided; the patient can cough and sneeze with their mouth open. Warm saline mouth rinses should be used to maintain cleanliness and hygiene at the operating site. Inhaling cannabis and smoking is also not permitted. The patient was instructed to use mouthwash containing 0.2% chlorhexidine digluconate for two weeks, and augmentin (625 mg) was prescribed along with Aceclofenac (100 mg) every eight hours for three days and oxymetazoline hydrochloride (0.05%) every 12 hours for five days as a nasal decongestant. The rehabilitation was planned by the use of a fixed partial denture prosthesis once the complete healing has occurred.

## Discussion

The term "oroantral fistula" refers to an epithelium-lined canal that may also comprise polyposis of the sinus membrane or granulation tissue and is most typically caused by iatrogenic oroantral communication. The proximity of the bicuspid apices and molars to the antrum makes the extraction of maxillary posterior teeth the most probable cause of OAF. Alternately, OAF may develop as a result of imprecise surgical planning while preparing the bone for the placement of a dental implant. When there is no infection, the majority of minor wounds with a diameter of 1-2 mm naturally heal. A secondary surgical intervention is necessary when persistent oroantral fistula defects are broader than 5 mm and have existed for over three weeks [2].

Patients with OAC or OAF may not exhibit any symptoms, but the majority report experiencing symptoms, which may be acute or long-term. Subjective and objective findings are typically used to make the clinical diagnosis. Epistaxis, the passage of liquid or air via the OAC or OAF, discomfort throughout and around the infected sinus tract, voice changes, and wheezing when speaking are examples of acute symptoms. Chronic symptoms comprise diminished pain, free fluid drainage through the oral fistula, mucopurulent nasal discharge, the likelihood of later-stage detection of antral polyps through the defect, postnasal drip, unpleasant intraoral taste and odor, voice changes, and ear pain [6]. All maxillofacial surgeons face a daunting task in achieving complete and long-term closure of the OAF. When deciding on the best way to close an OAF, several parameters should be evaluated, including the size of the defect, the anatomic area, the time since it was developed, infection, sinus inflammation, and the presence of foreign bodies.

The surgical management of OAC should adhere to and take into account two fundamental concepts. The primary rule is that the sinuses should have sufficient nasal outflow in order to keep out any form of infection. The second rule is that there should be no tension in the closure and that it should have a large foundation of soft tissue to ensure that a rich, vascularized flap can cover underlying, intact bone [7]. There are various drawbacks when it comes to other surgical procedures; the buccal advancement flap poses the problem of reduced vestibular depth, which hinders prosthetic rehabilitation. The palatal flap may pose a threat to greater palatine vessels and secondary donor site morbidity. The technique we employed in our case doesn’t mandate any flap elevation, thereby securing adequate vestibular depth; being less invasive, it gives better patient compliance [8].

In our case, we used PRF as it offers a broad array of merits. PRF is a natural matrix composed of various cell types that aid in angiogenesis, improved immune responses, and epithelial coverage; as a result of these properties, it can be used to accelerate tissue regeneration and improve healing. The angiogenic property of PRF has been attributed to the presence of cytokines in the fibrin matrix, which provides functional and structural stability, assisting in tissue regeneration [5]. Despite the multitude of usages of PRF in literature, there are only a few articles where PRF has been used for oroantral fistulas. Following the extraction of maxillary posterior teeth measuring around 5 mm in diameter, Gulsen used platelet-rich fibrin to treat 20 cases of oroantral communication. In one of the individuals, six blood clots were employed, and PRF was attached to the gingiva with non-resorbable sutures [9].

An oroantral fistula occurs when a rupture in the maxillary sinus's floor and epithelial membrane causes a blood clot to escape from an extraction socket. In the absence of the blood clot, the wound's healing epithelial cells fail to migrate from the periphery to the center, acting as a "scaffold" to develop on. Agarwal et al. used PRF with four blood clots; three were squeezed to form membranes, which were further rolled to form a PRF plug, and one PRF membrane was used to cover the PRF plugs, and the membrane was secured using horizontal mattress suturing for delayed management of OAC [10]. For the immediate closure of OAC, R. Pandikanda et al. [11] used the composite layer closure technique with PRF and collagen and PRF clots in the form of a membrane without any flap. The PRF-collagen mixture was sandwiched between two PRF membranes, and the membrane was secured using the horizontal mattress suturing technique. By the conclusion of the second month, the patients had not shown any signs of sinusitis. In every case, the vestibular depth was preserved after surgery.

Kani Bilginaylar used PRF clots for immediate closure of acute OACs after extraction of posterior maxillary molars in 21 patients with perforations larger than 3 mm in diameter, stating that the use of PRF enables the closure of OACs without primary flap closure or any other surgical intervention, making it less traumatic and easier [12].

The primary objective of OAF treatment is to achieve total closure and establish a seal between the maxillary sinus and the oral cavity. Flap closure has a number of disadvantages, including the requirement for a second operation, seal breaking, and wound dehiscence. In the investigation by Demetoglu et al., the OAF opening was fixed using simply a PRF membrane; no flap was raised, stating that it has a low likelihood of complications. The plasma-rich fibrin approach is a quick and efficient way to treat OACs with a diameter of 5 mm or less [13].

The periocol GTR is made up of porcine type I and III collagen fibers with no organic or chemical components, and it has a bilayer structure with a compact and porous layer. The membrane's compact layer has a smooth and condensed surface that prevents connective tissue infiltration, whereas the porous layer allows for cellular invasion [14,15]. The GTR membrane intervenes in the formation of soft tissue and prevents its ingrowth into the bony defect, thereby aiding in faster closure of the osseous defect and acceleration of bone formation [3].

As for us, we have used the sandwiching of the PRF membrane between the Schneiderian membrane along the overlying bone and the periocol GTR membrane without any flap advancements for closure of the oro-antral fistula. The membranes are then secured to the adjacent buccal and palatal mucosa using non-resorbable 3-0 BBS sutures. The tooth was replaced by a fixed partial denture. The use of PRF and collagen membrane allows minimal tension at the suture site, thereby improving the outcome and healing. The GTR membranes are anticipated to have a variety of qualities that, when coupled well, would result in a good material. Bioactive membrane functions have recently been tested in an effort to better promote tissue regeneration. In order to meet these high standards, future studies must examine the interaction of growth factors and antibacterial agents in barrier membranes, as well as the dynamics of material degradation. This procedure has advantages in terms of time and financial benefits, as well as reduced discomfort for the patient both before and after surgery, considering donor site intervention is not required. This approach also results in both hard tissue and soft tissue closure, as opposed to buccal sliding flap and palatal flap techniques, which exclusively produce soft tissue closure.

Sinus lift surgeries, which require an inflammation-free maxillary sinus, would not take place in the presence of an OAC, as it typically causes severe, persistent inflammation and thickens the sinus membrane. A substantial or greater risk of harm, particularly to the sinus membrane and oral mucosa, exists during sinus augmentation, which may arise from the soft tissue closure of an oroantral defect without bone height restoration before the placement of an implant. The autogenous graft-performed sinus closure paves the way for the subsequent traditional sinus lifting. For the closure of the oroantral defect, osseous support from soft tissue flap regeneration will be sufficient and a valid alternative. This technique allows for successive conventional sinus surgeries while protecting the teeth next to oroantral defects [7].

Implant placement in proximity to the maxillary sinus poses a great challenge, as any displacement into the sinus can cause complications. A disturbance in the anatomy and function of the clearance of mucous has been noticed. The bone graft material may be scattered, thereby blocking the ostium and further worsening the congestion or causing sinusitis which may accentuate the reformation of the oroantral communication, rendering failure of implant placement. A perforation in the sinus during the implant placement may lead to infection and peri-implantitis [16]. In our case, since the thickness of the new bone growth is less than the required bone levels for implant placement, it will render less primary stability. We have to incorporate a bone graft followed by implant placement if the conditions are optimal.

Jae Ha Baek et al. [17] intended to provide a sinus bone grafting technique using bone tacks, a collagen membrane, and an allograft as a therapy for OAC closure. An absorbable membrane comprised of a bag was applied during the process. Using this membrane as a wall to close the significant rupture in the sinus membrane. The membranes were fastened using bone tacks. The absorbable membrane was then reapplied after the allograft was filled into the maxillary sinus. By making a periosteum-releasing incision and using a tension-free suture, the primary closure was accomplished. After six months, adequate bone dimensions were obtained without the recurrence of OAC or maxillary sinusitis.

## Conclusions

The sandwich approach demonstrates good clinical regeneration and integration into the hard and soft tissues, and it is quick and simple. Relatively less pain and swelling develop following the surgery, and the vestibular architecture is very well preserved. This procedure is a new approach, and hence very limited literature is present. Here we present the case of oroantral fistula, which has been dealt with by the novel flapless system using the PRF sandwiched between the sinus lining and collagen membrane allowing for an immediate tension less closure. Through the use of this approach, the regenerated bone tissue can also accommodate an endosseous implant.

## References

[1] Khandelwal P, Hajira N (2017). Management of oro-antral communication and fistula: various surgical options. World J Plast Surg.

[2] Borgonovo AE, Berardinelli FV, Favale M, Maiorana C (2012). Surgical options in oroantral fistula treatment. Open Dent J.

[3] Al-Juboori MJ, Al-Attas MA, Magno Filho LC (2018). Treatment of chronic oroantral fistula with platelet-rich fibrin clot and collagen membrane: a case report. Clin Cosmet Investig Dent.

[4] Hafez WK, Seif SA, Shawky H, Hakam MM (2015). Platelet rich fibrin as a membrane for coverage of immediate implants: case-series study on eight patients. Tanta Dent J.

[5] Parvini P, Obreja K, Sader R, Becker J, Schwarz F, Salti L (2018). Surgical options in oroantral fistula management: a narrative review. Int J Implant Dent.

[6] Azzouzi A, Hallab L, Chbicheb S (2022). Diagnosis and management of oro-antral fistula: Case series and review. Int J Surg Case Rep.

[7] Koppolu P, Khan TA, Almarshad A. AA, Lingam AS, Afroz MM, Alanazi HF (2022). Management of a 20-year-old longstanding oroantral fistula: A case report and review of literature. Niger J Clin Pract.

[8] Balakrishnan Balakrishnan, Vijay Ebenezer, Prakash Prakash (2020). Buccal & palatal advancement flap in post extraction. Eur J Mol Clin Med.

[9] Gülşen U, Şentürk MF, Mehdiyev İ (2016). Flap-free treatment of an oroantral communication with platelet-rich fibrin. Br J Oral Maxillofac Surg.

[10] Agarwal B, Pandey S, Roychoudhury A (2016). New technique for closure of an oroantral fistula using platelet-rich fibrin. Br J Oral Maxillofac Surg.

[11] Pandikanda R, Singh R, Patil V, Sharma M, Shankar K (2019). Flapless closure of oro-antral communication with PRF membrane and composite of PRF and collagen - a technical note. J Stomatol Oral Maxillofac Surg.

[12] Bilginaylar K (2018). The use of platelet-rich fibrin for immediate closure of acute oroantral communications: an alternative approach. J Oral Maxillofac Surg.

[13] Demetoglu U, Ocak H, Bilge S (2018). Closure of oroantral communication with plasma-rich fibrin membrane. J Craniofac Surg.

[14] Elgali I, Omar O, Dahlin C, Thomsen P (2017). Guided bone regeneration: materials and biological mechanisms revisited. Eur J Oral Sci.

[15] Manuel S, Bonanthaya K, Panneerselvam Panneerselvam (2021). Oroantral communications and oroantral fistula. https://link.springer.com/chapter/10.1007/978-981-15-1346-6_24.

[16] An JH, Park SH, Han JJ, Jung S, Kook MS, Park HJ, Oh HK (2017). Treatment of dental implant displacement into the maxillary sinus. Maxillofac Plast Reconstr Surg.

[17] Baek JH, Kim BO, Lee WP (2021). Implant placement after closure of oroantral communication by sinus bone graft using a collagen barrier membrane in the shape of a pouch: A case report and review of the literature. Medicina (Kaunas).

